# Acute Post-stroke Hemiparkinsonism and Hemiparesis: A Unique Case with Successful Therapy

**DOI:** 10.7759/cureus.4950

**Published:** 2019-06-20

**Authors:** Raguraj Chandradevan, Ian H Rutkofsky, Bryan Lynn, Felisha L Kitchen, Marcus L Simmons

**Affiliations:** 1 Internal Medicine, Coliseum Medical Centers, Macon, USA; 2 Psychiatry, Coliseum Medical Centers, Macon, USA; 3 Internal Medicine, Mercer University, Macon, USA; 4 Obstetrics and Gynecology, Coliseum Medical Centers, Macon, USA

**Keywords:** stroke, vascular parkinsonism, hemiparesis, levodopa, basal ganglia

## Abstract

The diagnosis of a new onset movement disorder after a stroke has important clinical implications. The early assessment and timely diagnosis of post-stroke disorders is essential for influencing long-term outcomes. Localizing lesions and determining the underlying etiology is vital in targeting appropriate therapy. New and sudden onset of hemiparkinsonism with hemiparesis, rigidity, and tremor following an acute ischemic stroke is described here. This presentation was clinically diagnosed as acute post-stroke parkinsonism (APSP). The patient’s level of impairment was significant enough to compromise his activities of daily living (ADL), physical therapy (PT), and occupational therapy (OT) in an inpatient rehabilitation center. In the inpatient rehabilitation center, the patient received a trial of levodopa for suspected APSP. After levodopa therapy was initiated, we observed an improvement of his parkinsonian features with a sustained response and reached the conclusion that the clinical recognition of post-stroke parkinsonism treated with a targeted trial with levodopa may improve the quality of life. Proper treatment of APSP has the potential to provide the best opportunity for recovery and positively influence the long-term outcomes in similar patients.

## Introduction

Movement disorders can occur secondary to a variety of underlying diseases, be idiopathic, or as part of a genetic syndrome [1]. Cerebrovascular disease has been credited to cause up to 22% of secondary movement disorders. Acute post-stroke parkinsonism (APSP), a reported subsection of cerebrovascular disease, occurs after 1%-4% of strokes [2]. APSP is a well-described entity that presents with variable timing post-stroke [3]. The timing of the presentation of APSP is varied, with some cases presenting immediately after an acute stroke, whereas others display a progressive course, with symptoms developing later. These movement disorders have been frequently encountered in patients with ischemic and hemorrhagic strokes, cerebrovascular malformations, and dural arteriovenous fistulas affecting the basal ganglia, their connections, or both.

Movement disorders have a variety of clinical presentations according to the stroke lesion and their temporal relationships. Moreover, the predominant stroke symptoms may cloud the clinical picture, making proper diagnosis difficult. Vascular parkinsonism (VP) is a clinical phenomenon that usually refers to an isolated lacunar stroke in the basal ganglia and usually presents as bilateral lower body parkinsonism. In clinical practice, new onset of hemiplegic stroke and upper and lower extremity parkinsonism, contralateral to the hemiplegic side, has been documented, though it is rare. Because such symptomology is uncommon and associated with substantial co-morbidities, APSP has proven difficult to diagnose clinically and is easily misdiagnosed. Practitioners must always suspect post-stroke parkinsonism in patients that develop rigidity, akinesia, motor slowing, and gait impairment after a cerebrovascular accident [4-5].

Medical treatment for post-stroke movement disorders is similar to primary movement disorders since they share common underlying etiologies. The knowledge of pharmacologic treatment is still inadequate for many of these disorders, including post-stroke parkinsonism. The treatment mainly targets dopaminergic and GABAergic systems since they are believed to be the main pathways involved in post-stroke disorders [6]. The decision regarding the management of post-stroke movement disorders should be individualized and based on each clinician’s experience. Levodopa is the most effective drug for the symptomatic treatment of Parkinson’s disease, particularly in cases of intrusive or troublesome bradykinesia. In Parkinson’s disease, treatment should begin with small doses, such as carbidopa/levodopa 25/100 mg, one-half tablet two-three times daily with meals. Tolerance is usually assessed individually because of the increased susceptibility to psychiatric side effects. The smaller doses are slowly titrated to produce a useful clinical response. Patients often respond quickly, generally within days to weeks, depending upon symptom severity and titration schedule. With higher doses, clinicians should monitor for common and early side effects, such as nausea, dizziness, and headaches, and monitor for serious adverse effects like orthostatic hypotension or hypertension, in which case, tapering down may be indicated.

For patients with lacunar infarcts of the basal ganglia, the response to levodopa alone may be different [7]. The effect of responsive treatment with levodopa for vascular parkinsonism may be poor and short-lasting [8]. In these cases of post-stroke parkinsonism, a clinician may overlook a trial of levodopa and assume a poor response [9]. However, a positive response to levodopa in APSP should always be expected. This response can be explained by the presence of a remaining pool of striatal dopaminergic nerve endings in a dysfunctional nigrostriatal pathway that is sufficient to replenish the intrinsic dopaminergic needed from exogenous levodopa. Poor responders are those who have a significant dysfunction of the basal ganglia [7]. Our rare case is worth describing because such a presentation of hemiparkinsonism and hemiparesis is rare and underreported. This presentation sometimes may hinder the clinical diagnosis of APSP. Recognizing and treating this condition has the potential to improve quality of life.

## Case presentation

A 64-year-old right-handed man with left-sided hemiparesis was referred to a rehabilitation center for physical therapy (PT) and occupational therapy (OT). His medical history was significant for a 20-year history of hypertension, a 15-year history of insulin-requiring diabetes mellitus, coronary artery disease, and hyperlipidemia. He resides at an assisted living facility where he fell getting out of bed. The patient’s daughter reports that she spoke with him on the phone approximately one hour past the time of the event. As he explained to her over the phone about his weakness, she noticed that his speech was “off,” so she immediately picked him up and brought him to the emergency room for further evaluation. It is unknown if his fall was secondary to weakness or if his weakness developed secondary to his fall.

**Figure 1:**
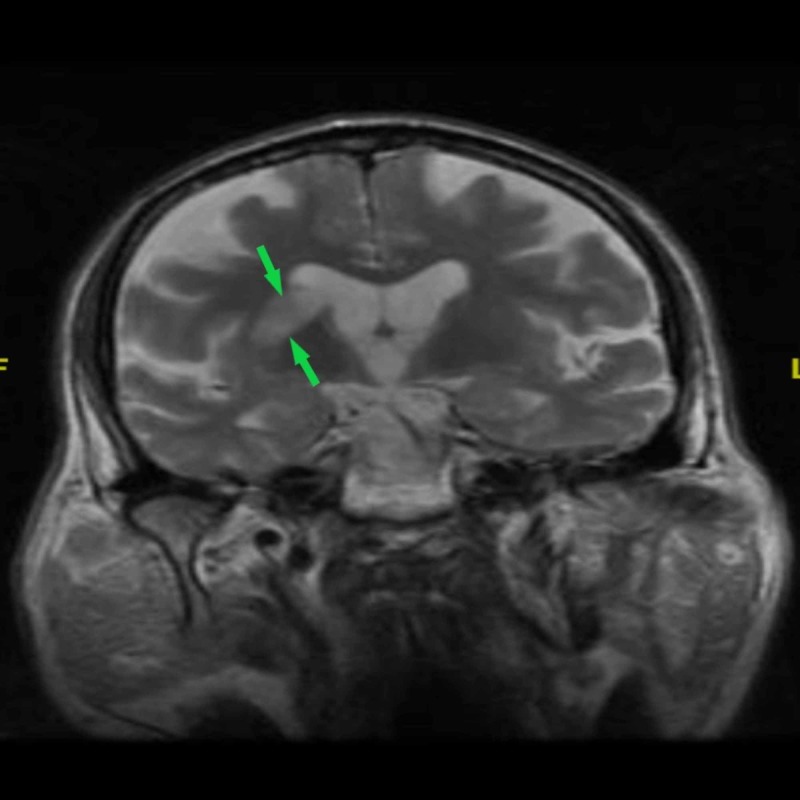
Brain MRI showing right corona radiata and right basal ganglia ischemic lesion (green arrows) in T2-weighted MRI (coronal plane) MRI: magnetic resonance imaging

**Figure 2:**
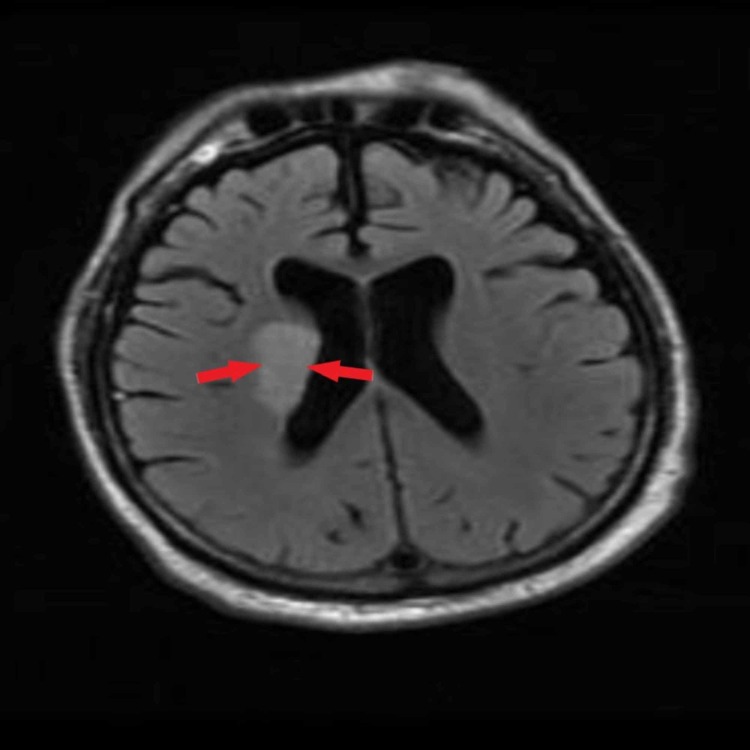
Brain MRI showing right corona radiata and right basal ganglia ischemic lesion (red arrows) in T2-weighted MRI (axial plane) MRI: magnetic resonance imaging

The patient’s presentation during admission showed left-sided hemiparesis with left-sided facial droop. Examination findings were consistent with left-sided lower extremity, upper extremity weakness, and left-sided facial asymmetry. The patient also had expressive aphasia. According to documentation, the patient showed signs of left-sided upper motor neuron (UMN) type facial nerve palsy and left-sided upper and lower motor limb strength graded 0/5. Rigidity was noted in the right upper and lower extremity with predominance over the right lower limb. The upper limb presented with a pronounced resting tremor observed over the right hand. On imaging, his head computed tomography (CT) was negative for an acute hemorrhagic process, which excluded acute and chronic bleeding or mass formation. Since the onset of weakness was indeterminate, as the patient’s weakness began upon waking from the bed, the ED physician on call could not estimate the therapeutic window for thrombolytic therapy, leaving him a poor candidate for treatment with thrombolytic tissue plasminogen activator (tPA). During further evaluation, his magnetic resonance imaging (MRI) brain showed acute ischemic cerebrovascular accident (CVA) of the right corona, caudate body, and right basal ganglia (Figures 1-2). Carotid ultrasound findings were insignificant for stenosis and electrocardiogram (EKG) revealed sinus rhythm with first degree AV block. His medical management continued with high dose aspirin, statins, and insulin.

His presentation stabilized at the ED. He was expected to be discharged back home to his assisted living facility to be managed with home health nursing, a nurse's aide, physical therapy (PT), occupational therapy (OT), and speech therapy (ST). The hospital team and his family decided his care should continue at the hospital, and he was transferred to the acute rehabilitation unit to continue his recovery. His evaluation at the rehabilitation unit revealed that he required to learn adaptive strategies in therapy to overcome the barriers caused by hemiparesis on his left non-dominant side. During further evaluation, his new-onset rigidity and resting tremor were further discussed, and he stated that he never had rigidity, postural instability, tremor, or bradykinesia any time prior to the new onset of left-sided weakness. We found that his rigidity was impairing him to the point to where he could not actively participate in PT. We clinically diagnosed him with acute post-stroke parkinsonism and added carbidopa and levodopa. We continued to follow him medically and work with him for his functional recovery as his rigidity highly impaired his PT and OT. Initially, we started the dose of 100 mg levodopa (levodopa/carbidopa) 25/100mg per oral tabs three times a day. He was assessed continuously and followed clinically. We noticed a marked improvement of his resting tremor first, followed by an improvement of his rigidity. With this dosage, he developed substantial improvement of his tremor in a week, but his rigidity did not improve. We decided to increase his medications to 100 mg levodopa (levodopa/carbidopa) 25/100mg per oral tabs four times per day. With the increased dosage of levodopa administration, we recognized a definite improvement in his rigidity in a week. This improvement helped him to further enhance his ADL, PT, and OT. As treatment continued beyond six months, during the follow-up, we observed a progressive improvement in his stiffness and rigidity on the right side, as did we see his motor strength improve from 0/5 to 2/5 in his shoulder flexors and hip flexors on the left non-dominant side.

## Discussion

Movement disorders occur uncommonly in post-stroke adults [10]. A study of the Lausanne stroke registry, with 2,500 first stroke patients found that 1% developed a movement disorder [11]. New onset involuntary movements after a stroke is an important consideration when evaluating patients. Post-stroke movement disorders are defined as paroxysmal, recurrent, transient, or as a delayed, permanent syndrome [8]. Thus, this kind of clinical manifestation can be considered a distinct clinical entity. A wide variety of clinical presentations, including tremor, chorea-ballism, dystonia, athetosis, myoclonus, ataxia, and parkinsonism, have been described [2,10].

Parkinsonism is defined as a complex syndrome with many etiologies. Parkinson’s disease (PD) is the second most common neurodegenerative disease in the elderly population. Despite the high prevalence of Parkinson's disease, the etiology remains unknown and is often represented as idiopathic Parkinson’s disease for about 75% of all cases. Vascular parkinsonism (VP) is a type of parkinsonism secondary to cerebrovascular disease [12]. In population cohort studies, it accounts for 2.5%-12% of all cases of parkinsonism [13]. VP has been associated with unilateral or bilateral basal ganglia infarcts in the striatum or lentiform nucleus. Numerous studies have shown that vascular lesions disrupting the interconnecting fiber tracts between basal ganglia, thalamus, and motor cortex can lead to the disruption of sensory-motor integration as well as descending reticular pathways to the major centers of the brain stem. Two forms of VP may be suggested: one with acute onset, related to basal ganglia infarcts, as seen in our case, or VP with slow progression, possibly associated with white matter ischemia [14]. Vascular parkinsonian features usually occur in the contralateral side of the lesion in the basal ganglia. Nevertheless, there have been instances recorded of a phenomenon called “cerebral diaschisis,” where the misleading nature of the measured cortical metabolic asymmetry has been well-explained in acute ischemic stroke [15]. Furthermore, studies have reported that the distribution of deficit can be ipsilateral/contralateral to the motor weakness or bilateral with predominance in the lower limb [16].

Clinicians should consider the possibilities of movement disorders, especially VP, when treating acute stroke patients in clinical practice. In the clinical course, most post-stroke movement symptoms are transient and self-limiting. However, a proper diagnosis with a treatment strategy is essential because, sometimes, these might persist and become life-threatening, especially with progressive neurological disorders with a significant decrement in quality of life. Timely diagnosis and treatment could hasten the recovery and prevent the worsening of disease progression. Although there are no established treatment guidelines based on randomized clinical trials reflecting the prevalence of post-stroke movement disorders, we recommend such a study. The lack of such an investigation into this clinical entity reflects the low prevalence/recognition of post-stroke movement disorders. The treatment strategies include controlling dopaminergic excitability and anticholinergic drugs. Vascular parkinsonism has been considered a non-responder or poor responder to treatment [17]. However, observational clinical trials performed with acute onset vascular parkinsonism documented that L-dopa treatment induced an excellent response in the majority of patients. Nevertheless, the literature has documented that patients with Parkinson’s disease using levodopa treatment can have an increase in serum homocysteine levels [18]. An increase in the homocysteine levels may alter cerebral blood flow velocities and resistances [18], which can further compromise the vascular flow in a patient who had a cerebrovascular event. This evidence suggests that if the short-term treatment of levodopa is implemented, clinical and laboratory evaluations are necessary to follow throughout its treatment.

## Conclusions

This elderly gentleman with an ischemic stroke presented with the classic features of acute onset parkinsonism. His parkinsonism was contralateral to his vascular cerebral lesion and may be due to the involvement of the lower limbs responsible for gait difficulty and postural instability. He substantially improved his parkinsonism features within a short-time; most notably, an improvement in first his tremor and then his rigidity.

For individuals with post-stroke movement disorders, it is crucial to localize the lesions to hypothesize the underlying etiology. These patients may need targeted therapy, which can importantly contribute to the disability and long-term outcome. Our patient exhibits a unique presentation of vascular parkinsonism, following an acute basal ganglia infarction. The course of ischemic change or lesion affecting the pallido- or nigro-thalamic pathways can interrupt the basal ganglia output to the thalamic ventral-lateral and ventral-anterior nuclei and present with the unilateral features of Parkinson’s disease on the same side of the basal ganglia infarction. A possible explanation could be ipsilateral thalamic diaschisis, which is opposite to the motor deficit. Individuals with multiple co-morbidities may present with extrapyramidal symptoms after a stroke, but may not be recognized and often mistaken for psychomotor slowing. Our patient’s dramatic improvement in parkinsonian features and motor deficit, with sustained response to levodopa therapy, warrants further attention. Furthermore, we recommend a trial of levodopa therapy for basal ganglia strokes when parkinsonian features are present on neurological examination.
